# Detection of circulating tumour DNA after neoadjuvant chemoradiotherapy in patients with locally advanced oesophageal cancer

**DOI:** 10.1002/path.6016

**Published:** 2022-10-31

**Authors:** Ben M Eyck, Maurice PHM Jansen, Bo Jan Noordman, Peggy N Atmodimedjo, Berend J van der Wilk, John WM Martens, Jean A Helmijr, Corine M Beaufort, Bianca Mostert, Michail Doukas, Bas PL Wijnhoven, Sjoerd M Lagarde, J Jan B van Lanschot, Winand NM Dinjens

**Affiliations:** ^1^ Department of Surgery, Erasmus MC Cancer Institute University Medical Centre Rotterdam Rotterdam The Netherlands; ^2^ Department of Medical Oncology, Erasmus MC Cancer Institute University Medical Centre Rotterdam Rotterdam The Netherlands; ^3^ Department of Pathology, Erasmus MC Cancer Institute University Medical Centre Rotterdam Rotterdam The Netherlands

**Keywords:** circulating tumour DNA, oesophageal cancer, neoadjuvant therapy, residual neoplasm, active surveillance, oesophagectomy, high‐throughput nucleotide sequencing, polymerase chain reaction

## Abstract

Active surveillance instead of standard surgery after neoadjuvant chemoradiotherapy (nCRT) has been proposed for patients with oesophageal cancer. Circulating tumour DNA (ctDNA) may be used to facilitate selection of patients for surgery. We show that detection of ctDNA after nCRT seems highly suggestive of major residual disease. Tumour biopsies and blood samples were taken before, and 6 and 12 weeks after, nCRT. Biopsies were analysed with regular targeted next‐generation sequencing (NGS). Circulating cell‐free DNA (cfDNA) was analysed using targeted NGS with unique molecular identifiers and digital polymerase chain reaction. cfDNA mutations matching pre‐treatment biopsy mutations confirmed the presence of ctDNA. In total, 31 patients were included, of whom 24 had a biopsy mutation that was potentially detectable in cfDNA (77%). Pre‐treatment ctDNA was detected in nine of 24 patients (38%), four of whom had incurable disease progression before surgery. Pre‐treatment ctDNA detection had a sensitivity of 47% (95% CI 24–71) (8/17), specificity of 85% (95% CI 42–99) (6/7), positive predictive value (PPV) of 89% (95% CI 51–99) (8/9), and negative predictive value (NPV) of 40% (95% CI 17–67) (6/15) for detecting major residual disease (>10% residue in the resection specimen or progression before surgery). After nCRT, ctDNA was detected in three patients, two of whom had disease progression. Post‐nCRT ctDNA detection had a sensitivity of 21% (95% CI 6–51) (3/14), specificity of 100% (95% CI 56–100) (7/7), PPV of 100% (95% CI 31–100) (3/3), and NPV of 39% (95% CI 18–64) (7/18) for detecting major residual disease. The addition of ctDNA to the current set of diagnostics did not lead to more patients being clinically identified with residual disease. These results indicate that pre‐treatment and post‐nCRT ctDNA detection may be useful in identifying patients at high risk of disease progression. The addition of ctDNA analysis to the current set of diagnostic modalities may not improve detection of residual disease after nCRT. © 2022 The Authors. *The Journal of Pathology* published by John Wiley & Sons Ltd on behalf of The Pathological Society of Great Britain and Ireland.

## Introduction

Worldwide, approximately half of patients with oesophageal cancer present with locally advanced disease and can undergo potentially curative treatment, mostly consisting of neoadjuvant chemoradiotherapy (nCRT) followed by surgical resection [1, 2]. After nCRT and surgery, one third of patients have a pathologically complete response in the resection specimen [3, 4, 5]. These patients could perhaps be treated curatively with nCRT alone, without undue exposure to the risks of surgery, including perioperative mortality of 1–5%, severe postoperative morbidity, and decreased health‐related quality of life [6, 7].

Currently, active surveillance is being investigated for patients without clinical evidence of residual disease after nCRT [8]. During this organ‐sparing approach, patients undergo clinical response evaluations and undergo surgery only in case of highly suspected or proven locoregional residual disease. Multiple invasive diagnostic modalities are used for detecting residual disease after nCRT, such as endoscopy with biopsies of the primary tumour location and endoscopic ultrasonography (EUS) with fine‐needle aspiration of suspected nodes, and positron emission tomography with computed tomography (PET/CT) for distant interval metastases. Individually, these modalities are inaccurate for detecting residual locoregional disease [9]. Even when combined, major residual disease (>10% residual tumour in the resection specimen) is not detected in 10% of patients [10]. If these residues remain undetected, unresectable regrowth or distant metastases may develop.

Circulating tumour DNA (ctDNA) is a less invasive and potentially accurate biomarker for monitoring residual disease. ctDNA consists of DNA fragments that are shed into the circulation by tumour cells and can be identified in blood plasma by molecular profiling. ctDNA analyses can be used for cancer diagnosis, assessment of treatment response, molecular tumour profiling, and as a prognostic biomarker [11]. It is a promising biomarker for evaluating treatment response in metastatic oesophageal cancer, as well as a promising prognostic biomarker after surgery for locally advanced oesophageal cancer [12, 13]. Whether ctDNA is a candidate marker for detecting locoregional residual disease after nCRT is unclear. We aimed to determine whether mutations found in pre‐treatment tumour biopsies could also be detected in pre‐treatment blood plasma of patients with locally advanced oesophageal cancer and whether it is feasible to correlate changes in ctDNA after nCRT with residual disease.

## Materials and methods

### Patients and samples

This study was approved by the Erasmus MC Medical Ethical Committee (MEC‐2013‐211/2017‐392) and was performed in accordance with the Declaration of Helsinki. Written informed consent was obtained from all patients.

**Figure 1:**
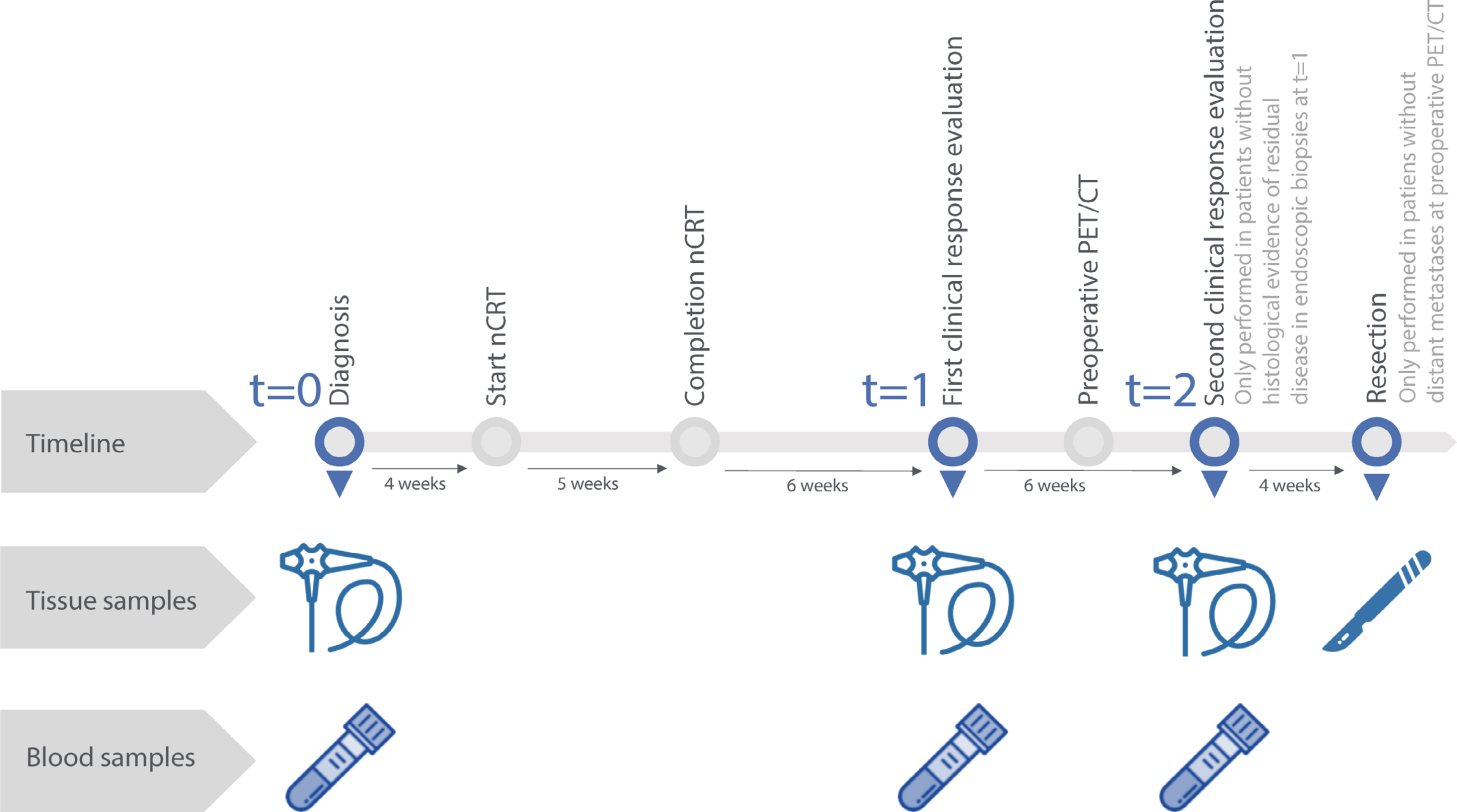
Overview of the study and sample collection before and after neoadjuvant chemoradiotherapy (nCRT). The icon of an endoscope represents a set of endoscopic biopsies, the icon of a scalpel represents the surgical resection specimen, and the icon of a blood tube represents a plasma sample.

Patients with locally advanced oesophageal cancer who were enrolled in the pre‐Surgery As Needed for Oesophageal cancer (preSANO) trial or the surgery arm of the Surgery As Needed for Oesophageal cancer (SANO) trial at the Erasmus MC (Rotterdam, The Netherlands) between 2016 and 2018 were eligible [8, 10]. Patients were approached for the present study directly after providing informed consent for the preSANO or SANO trial. Patients included in the preSANO trial provided separate informed consent. Patients included in the SANO trial could opt in for blood sampling on the informed consent form of the SANO trial. An overview of the present study and the sample collection is provided in Figure 1. Pre‐treatment staging consisted of endoscopy with biopsies, EUS, PET/CT, and diagnostic computed tomography (CT) of the neck, chest, and abdomen. All patients were scheduled to undergo nCRT according to the ChemoRadiotherapy for Oesophageal cancer followed by Surgery Study (CROSS) regimen [3]. This regimen consists of five weekly cycles of carboplatin (area under the curve 2 mg/ml per min) and paclitaxel (50 mg/m^2^) on the first day of each week with concurrent 41.4 Gy radiotherapy in 23 daily fractions of 1.8 Gy, followed by oesophagectomy. At the location of the primary tumour, at least four endoscopic bite‐on‐bite biopsies were taken before (*t* = 0) and at 6 weeks after completion of nCRT (*t* = 1). Patients with histologically proven residual tumour at *t* = 1 underwent PET/CT, and in cases where no distant interval metastases were found, surgical resection was performed. Patients without residual tumour at *t* = 1 underwent PET/CT, followed by another endoscopy with biopsies as well as EUS with fine‐needle aspiration of suspected nodes at 12 weeks after nCRT (*t* = 2). Subsequently, all patients without distant metastases underwent surgical resection, regardless of the presence of residual locoregional tumour.

Tissue samples from endoscopic biopsies and resection specimens were formalin‐fixed and paraffin‐embedded (FFPE) according to standard protocols. At *t* = 0, *t* = 1, and *t* = 2, within 7 days of endoscopic biopsy, 20 ml of blood was drawn into ethylenediaminetetraacetic acid (EDTA) tubes (BD Vacutainer®; BD, Plymouth, UK) in the preSANO trial and into CellSave preservation tubes (Menarini Silicon Biosystems Inc., San Diego, CA, USA) in the SANO trial. To provide stable ctDNA levels, plasma from EDTA tubes was isolated within 1 h, whereas plasma from CellSave tubes was isolated within 96 h, both by centrifuging at 1,711 × *g* for 10 min, followed by another 10 min at 12,000 × *g* [14]. Plasma samples were snap‐frozen and stored at −80 °C.

### DNA isolation and quantification

Tumour areas on haematoxylin‐stained 4‐μm FFPE sections of *t* = 0 endoscopic biopsies and resection specimens were marked by a GI pathologist (MD) and manually macrodissected under a dissecting microscope. DNA was extracted using proteinase K and a 5% Chelex resin. Circulating cell‐free DNA (cfDNA), i.e. the fraction of all tumour‐derived and non‐tumour‐derived circulating DNA fragments, was isolated from plasma samples using QIAamp Circulating Nucleic Acid Kits (Qiagen, Venlo, The Netherlands) or a customised Maxwell RSC cfDNA Plasma Kit (Promega, Madison, WI, USA). Concentrations of cfDNA were quantified using a Quant‐iT™ dsDNA High‐Sensitivity Assay Kit (Thermo Fisher Scientific, Landsmeer, The Netherlands) and Qubit® 2.0 fluorometer (Thermo Fisher Scientific). The DNA inputs and concentrations are listed in supplementary material, Table S1.

### Molecular profiling

Next‐generation sequencing (NGS) was performed using the Ion Torrent S5 GeneStudio Prime System (Thermo Fisher Scientific). For tissue‐derived DNA, a custom‐made pan‐cancer diagnostic NGS panel (Version 5.1, July 2017) with a mean read coverage depth of >1,000 reads was used. The composition of the panel has been reported previously and includes at least all regions covered by the NGS assay that was used for cfDNA [15]. For one patient, tumour whole‐exome sequencing data were already available and were used instead. For further analysis of tissue mutations, only pathogenic non‐synonymous single‐nucleotide variants, deletions, insertions, substitutions, and stop‐gains within the exome and splice site with a variant allele frequency (VAF) greater than 8% in tumour tissue were selected (which is the standard in our routine practice for molecular diagnostics of FFPE tissue‐derived DNA).

Twenty‐three patients had a *t* = 0 tissue mutation that was covered by the NGS assay used for cfDNA. NGS with unique molecular identifiers (NGS‐UMI) of cfDNA was performed using the Oncomine Colon cfDNA assay (Thermo Fisher Scientific). This assay covers 48 amplicons, evaluating 242 hotspot mutations for gastrointestinal cancers and novel mutations within the adenomateus polyposis coli (*APC*), RAC‐alpha serine/threonine‐protein kinase (*AKT1*), B‐Raf (*BRAF*), beta‐catenin (*CTNNB1*), epidermal growth factor receptor (*EGFR*), receptor tyrosine‐protein kinase erbB‐2 (*ERBB2*), F‐box/WD repeat‐containing protein 7 (*FBXW7*), GNAS complex locus (*GNAS*), K‐Ras (*KRAS*), dual specificity mitogen‐activated protein kinase kinase 1 (*MAP2K1*), N‐Ras (*NRAS*), phosphatidylinositol‐4,5‐bisphosphate 3‐kinase catalytic subunit alpha (*PIK3CA*), mothers against decapentaplegic homolog 4 (*SMAD4*), and tumour protein 53 (*TP53*) genes. The mean read coverage depth was >20,000. Sequencing data were processed using Torrent Suite and analysed using Variant Caller v5.10.0.18 (both Thermo Fisher Scientific). Variant annotation was performed using the ANNOtate VARiation (ANNOVAR) custom pipeline (http://openbioinformatics.org/annovar) in Galaxy (http://galaxyproject.org). Additional digital polymerase chain reaction (dPCR) of cfDNA was performed using the naica® Crystal PCR system (Stilla Technologies, Villejuif, France) in 14 patients for whom specific assays of *t* = 0 tissue mutations were available. Sequencing and dPCR data are provided in supplementary material, Tables S1 and S2. Five patients had a *t* = 0 tissue mutation that was not covered by the Oncomine NGS assay and for which no dPCR assay was available. These patients were excluded from further ctDNA analysis.

Mutations in *t* = 0 tissue‐derived DNA were compared with those in cfDNA to ensure that blood‐derived DNA mutations originated from tumour cells. By doing so, confounding mutations such as clonal haematopoiesis were excluded for the analysis [16]. Plasma samples were considered ctDNA‐positive if the specific *t* = 0 tissue mutation could be matched with the same specific mutation in cfDNA, detected by either NGS or dPCR. To eliminate false‐positive findings in ctDNA, only variants detected with three or more unique molecules by NGS or five or more copies by dPCR were considered reliable. Cut‐offs were assay‐specific and were determined in healthy blood donors (data not shown). Mutations in *t* = 0 tumour biopsies were also matched with mutations in the surgical resection specimen to investigate intratumoural heterogeneity before and after nCRT.

### Statistical analysis

Continuous variables were compared between ctDNA‐positive and ctDNA‐negative patients using Student's *t*‐test or the Kruskal–Wallis test in cases of non‐normal distribution. Categorical variables were compared using the *χ* ^2^ test or Fisher's exact test in cases of small expected cell counts or if two categorical variables were being compared. cfDNA concentrations and ctDNA VAF were correlated with patient and tumour characteristics using linear regression for continuous variables and the Kruskal–Wallis test for categorical variables. The sensitivity, specificity, positive predictive value (PPV), and negative predictive value (NPV) for discriminating major residual disease from minor residual disease after nCRT were calculated. Major residual disease was defined as Chirieac's tumour regression grade (TRG) 3 or 4 (i.e. >10% residual tumour) in the resection specimen or incurable disease progression before surgery [17]. Minor residual disease was defined as TRG 1 or 2 (i.e. ≤10% residual tumour) in the resection specimen. Two‐sided *p* < 0.05 was considered statistically significant. Analyses were performed using ‘tableone’, ‘stats’, ‘survival’, and ‘ggplot2’ packages in R version 3.6.1 (R Foundation for Statistical Computing, Vienna, Austria) [18, 19, 20].

## Results

### Patient characteristics

**Figure 2:**
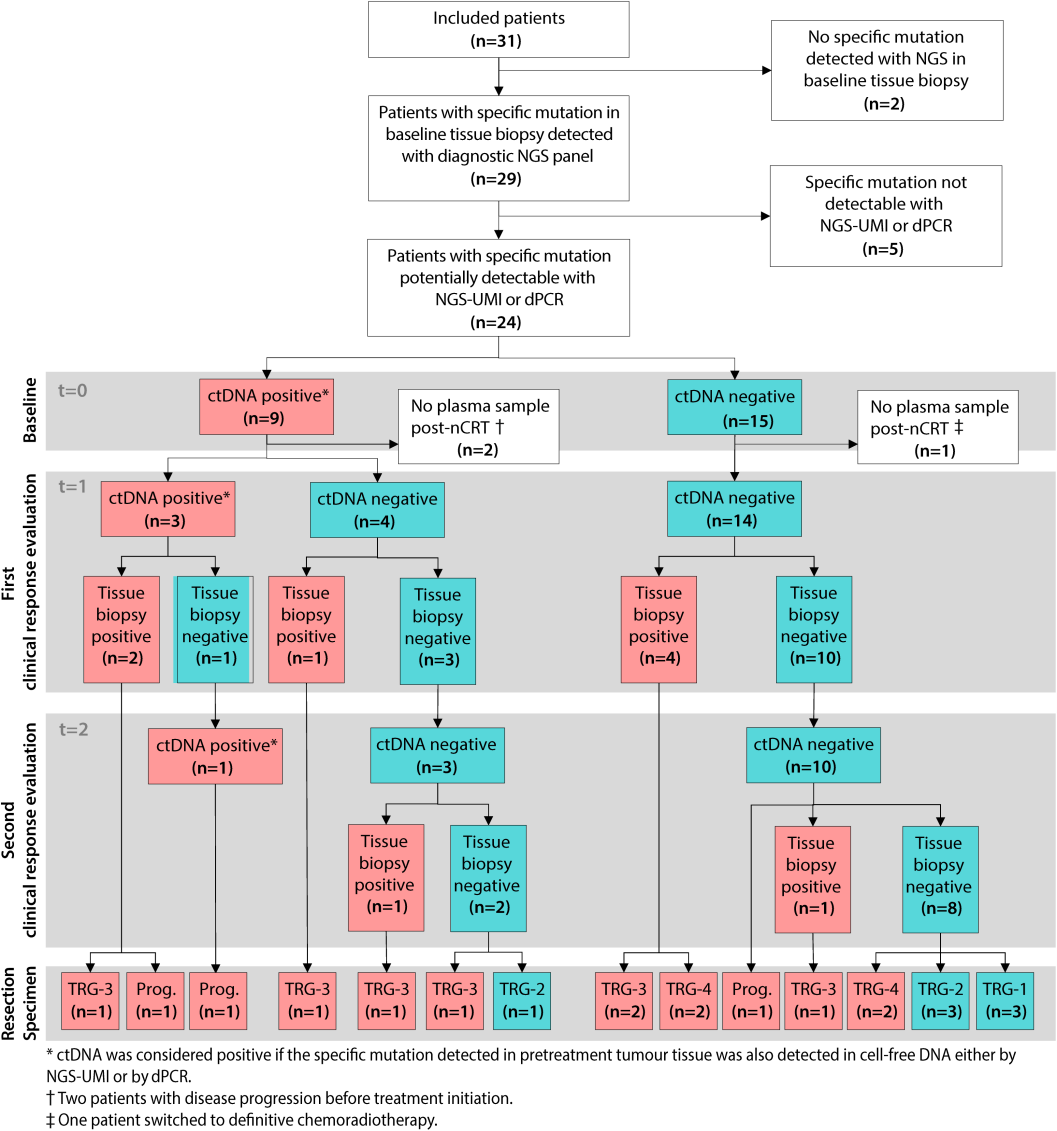
Flowchart of patients and samples in relation to detection of circulating tumour DNA (ctDNA). A red‐coloured box represents a positive sample and a blue‐coloured box represents a negative sample. ctDNA, circulating tumour DNA; nCRT, neoadjuvant chemoradiotherapy; NGS, next‐generation sequencing; Prog., no resection due to progression prior to surgery; TRG, tumour regression grade. TRG 1, 0%; TRG 2, 1–10%; TRG 3, 10–50%; TRG 4, >50% residual vital tumour cells.

Thirty‐one consecutive patients were selected for serial blood sampling, from whom a total of 79 plasma samples (31 at *t* = 0, 28 at *t* = 1, and 20 at *t* = 2) were collected. Of the 31 patients included, 28 (90%) underwent nCRT. One patient refused to undergo surgery and switched to definitive chemoradiotherapy before starting nCRT, whereas two patients had disease progression and started induction chemotherapy instead. Surgery was performed in 23 of 28 patients who underwent nCRT (82%). The baseline patient and tumour characteristics are summarised in Table 1. A flowchart of the patients in relation to ctDNA detection and pathological outcomes is shown in Figure 2.

**Table 1:** Baseline characteristics stratified by detection of circulating tumour DNA (ctDNA) in pre‐treatment plasma samples.

	Total	Pre‐treatment ctDNA‐positive	Pre‐treatment ctDNA‐negative	*p*
Patients, *n* ^†^	31	9	15	
Age (years), median (IQR)	70 (65–73)	71 (63–74)	70 (67–72)	0.61
Sex, *n* (%)				0.62
Female	5 (16)	1 (11)	4 (27)	
Male	26 (84)	8 (89)	11 (73)	
Histology, *n* (%)				1.00
Adenocarcinoma	27 (87)	8 (89)	13 (87)	
Squamous cell carcinoma	4 (13)	1 (11)	2 (13)	
Tumour location, *n* (%)*				1.00
Middle	4 (13)	1 (11)	2 (13)	
Distal	21 (68)	7 (78)	10 (67)	
Oesophagogastric junction	6 (19)	1 (11)	3 (20)	
cT stage, *n* (%)^‡^				0.26
2	6 (19)	0 (0)	4 (27)	
3	25 (81)	9 (100)	11 (73)	
cN stage, *n* (%)^‡^				0.30
0	11 (36)	1 (11)	6 (40)	
1	11 (36)	4 (44)	6 (40)	
2	8 (26)	3 (33)	3 (20)	
3	1 (3)	1 (11)	0 (0)	
Stage, *n* (%)^‡^				
II	14 (45)	1 (11)	9 (60)	**0.03**
III	17 (56)	8 (89)	6 (40)	
Differentiation, *n* (%)				0.15
Good/moderate (G1/G2)	22 (71)	5 (56)	12 (80)	
Poor/non (G3)	8 (26)	4 (44)	2 (13)	
Unknown	1 (3)	0 (0)	1 (7)	
Gross tumour volume (ml), median (IQR)	36.7 (30.1–70.1)	47.2 (36.7–97.5)	33.3 (27.5–36.9)	0.06
Tumour length (cm), mean (SD)	4.9 (3)	5.6 (3)	4.8 (3)	0.50
Neoadjuvant chemoradiotherapy, *n* (%)				
Yes	28 (90)	7 (78)	14 (93)	0.63
No	3 (10)	2 (22)^§^	1 (7)^∥^	
Surgery, *n* (%)				
Yes	23 (74)	5 (56)	13 (87)	0.22
No	8 (26)	4 (44)	2 (13)	

*P* values in bold are statistically significant.

* Tumours located in the proximal half of the oesophagus between the tracheal bifurcation and oesophagogastric junction are considered middle oesophageal tumours, whereas tumours located in the distal half of the oesophagus between the tracheal bifurcation and oesophagogastric junction are considered distal oesophageal tumours. Tumours invading both the oesophagus and the gastric cardia are considered junctional tumours.

^†^ The numbers of ctDNA‐positive and ctDNA‐negative patients do not add up to the total since two patients had no mutation in the baseline tumour tissue and five patients had a tumour tissue mutation that was not covered by the assay used for next‐generation sequencing with unique molecular identifiers or by digital PCR. These patients were excluded from the ctDNA analysis.

^‡^ According to the 7th edition of the Union for International Cancer Control *TNM Classification of Malignant Tumours* [21].

^§^ Two patients had progression before starting nCRT and started induction chemotherapy (carboplatin and paclitaxel) instead.

^∥^ One patient switched to definitive chemoradiotherapy (six instead of five weekly cycles of carboplatin and paclitaxel, with concurrent 50.4 Gy instead of 41.4 Gy radiotherapy) before starting nCRT because the patient did not want to undergo surgery.

### Pre‐treatment tissue mutations

**Figure 3:**
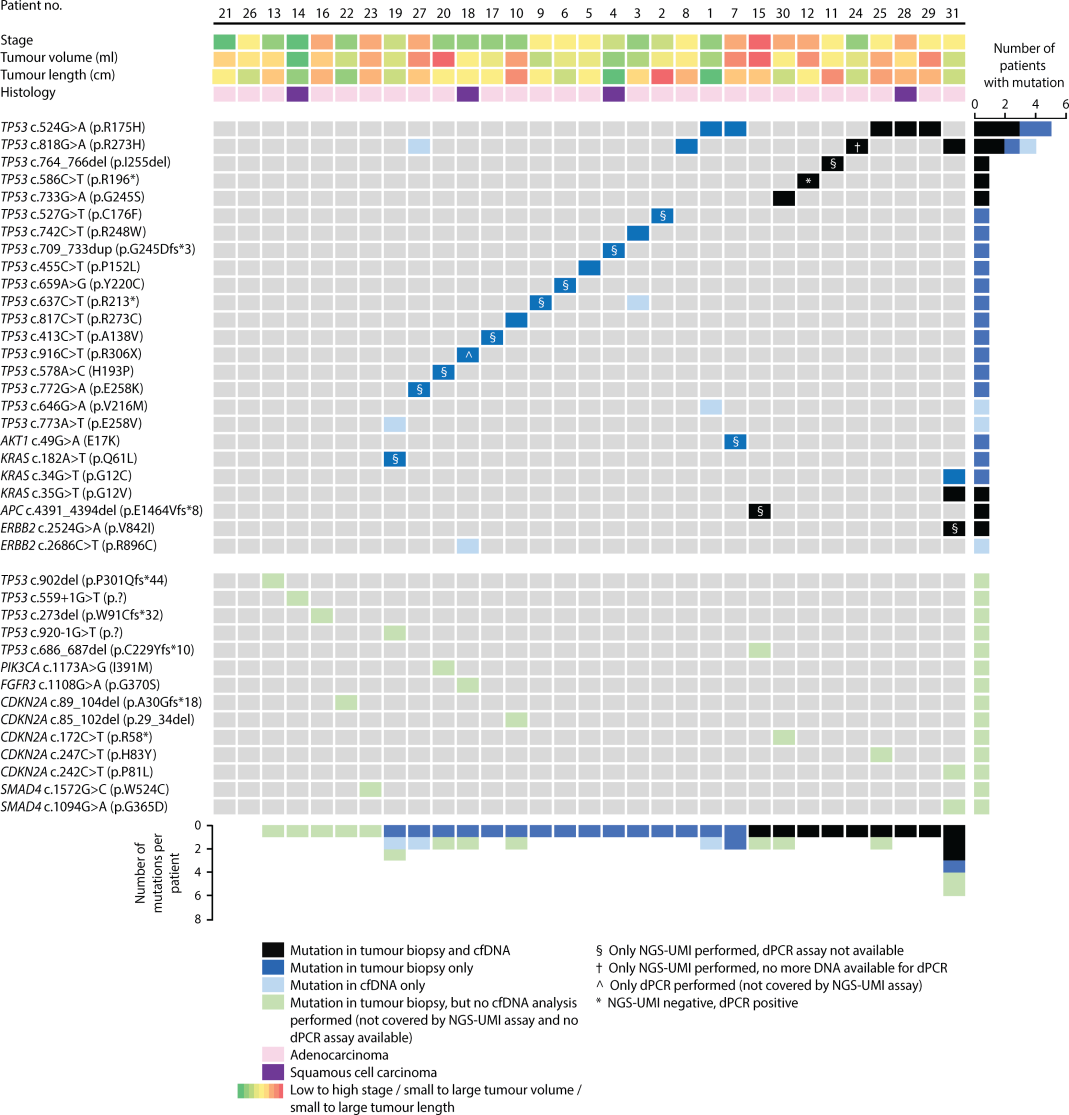
Mutations identified in pre‐treatment (*t* = 0) tumour biopsies and circulating cell‐free DNA (cfDNA) by next‐generation sequencing with unique molecular identifiers (NGS‐UMI) and digital polymerase chain reaction (dPCR).

In 29 of 31 patients (94%), a total of 42 tissue mutations were detected in *t* = 0 tumour biopsies with a median VAF of 53% [interquartile range (IQR) 42–68]. These 42 mutations consisted of 36 unique mutations, of which *TP53* p.R175H; c.524G>A was present in five different patients and *TP53* p.R273H; c.818G>A in three different patients. Mutations were found in *TP53* (27 patients), cyclin‐dependent kinase inhibitor 2A (*CDKN2A*) (five patients), *KRAS* (three patients), *SMAD4* (two patients), *APC* (one patient), *ERBB2* (one patient), *AKT1* (one patient), fibroblast growth factor receptor 3 (*FGFR3*) (one patient), and *PIK3CA* (one patient). Of the 36 unique mutations, 22 (61%) were non‐synonymous single nucleotide variants (SNVs), five (14%) were frameshift, two (6%) were non‐frameshift deletions, five (14%) were stop‐gains, and two (6%) were splice site mutations (Figure 3).

### Pre‐treatment ctDNA detection

In total, 24 patients had a *t* = 0 tissue mutation that was potentially detectable in cfDNA (13 patients by both NGS‐UMI and dPCR, ten patients by NGS‐UMI alone, and one patient by dPCR alone; supplementary material, Table S2). Median cfDNA input was 4.0 ng (IQR 4.0–18.9) for NGS‐UMI and 13.3 ng (IQR 9.3–19.0) for dPCR. In nine of 24 patients (38%) with a potentially detectable *t* = 0 tissue mutation, the same specific mutation was detected in blood plasma at *t* = 0 (Figures 2 and 3). Eight patients were ctDNA‐positive by NGS‐UMI, five of whom had dPCR assays available. All five were also ctDNA‐positive by dPCR. One patient was ctDNA‐negative by NGS‐UMI but positive by dPCR (supplementary material, Table S2). Of these nine ctDNA‐positive patients, seven had a *TP53* mutation, one had an *APC* mutation, and one had a *TP53*, *ERBB2*, and *KRAS* mutation. Eight mutations were non‐synonymous SNVs, two were deletions, and one was a stop‐gain variant. The most common mutations were *TP53* p.R175H; c.524G>A (three patients) and *TP53* p.R273H; c.818G>A (two patients) (Figure 3 and supplementary material, Table S2).

Of nine patients with pre‐treatment ctDNA detection, two had disease progression before treatment initiation and six had major residual disease after nCRT (of whom two had disease progression prior to surgery). None of these patients had TRG 1 (i.e. no residual tumour in the resection specimen). Of 15 patients without pre‐treatment ctDNA detection, six had minor residual disease after nCRT (of whom three had TRG 1) (Figure 2). Only one of these patients had progression prior to surgery. ctDNA was detected before treatment in nine of 16 patients with either disease progression prior to treatment initiation or major residual disease after nCRT. ctDNA was not detected before treatment in six of seven patients with minor residual disease. This resulted in a sensitivity of 47% (95% CI 24–71) (8/17), specificity of 85% (95% CI 42–99) (6/7), PPV of 89% (95% CI 51–99) (8/9), and NPV of 40% (95% CI 17–67) (6/15).

Disease stage was higher in ctDNA‐positive than in ctDNA‐negative patients (stage III: 89% versus 40%, *p* = 0.03). Gross tumour volume was 44.7 ml (95% CI 36.8–98.0) in ctDNA‐positive patients compared with 31.5 ml (95% CI 24.6–33.8) in ctDNA‐negative patients (*p* = 0.06). Other baseline patient and tumour characteristics were not significantly different between ctDNA‐positive and ctDNA‐negative patients (Table 1). cfDNA concentrations and ctDNA VAFs at *t* = 0 were not correlated with baseline tumour characteristics (supplementary material, Figure S1).

### Post‐treatment ctDNA detection

Of the 24 patients with a potentially detectable *t* = 0 tissue mutation, 21 (88%) had post‐nCRT (*t* = 1 and/or *t* = 2) plasma available (Figure 2). At *t* = 1, median cfDNA input was 20.0 ng (IQR 9.12–21.0) for NGS‐UMI and 17.6 ng (IQR 12.5–21.7) for dPCR. At *t* = 2, median cfDNA input was 20.0 ng (IQR 7.5–21.0) for NGS‐UMI and 21.3 ng (IQR 6.9–28.3) for dPCR.

**Figure 4:**
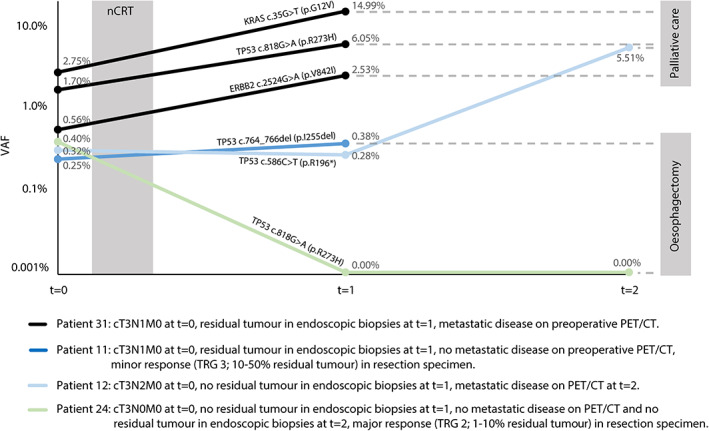
Four informative examples of typical patients with circulating tumour DNA (ctDNA) levels pre‐treatment (*t* = 0) and 6 weeks (*t* = 1) and 12 weeks (*t* = 2) after neoadjuvant chemoradiotherapy (nCRT) corresponding to outcomes of endoscopic biopsies and surgery. The dots represent the analysis of ctDNA. Percentages are variant allele frequencies (VAFs) of ctDNA measured by next‐generation sequencing with unique molecular identifiers (NGS‐UMI) in patients 11, 24, and 31, and by digital polymerase chain reaction (dPCR) in patient 12.

Three patients were ctDNA‐positive after nCRT. At *t* = 1, one patient (patient 11) had *TP53* c.759_761del p.I255del (NGS‐UMI VAF 0.38%, dPCR assay not available), one patient (patient 12) had *TP53* c.586C>T p.R196* (NGS‐UMI VAF 0.00%, dPCR VAF 0.28%), and one patient (patient 31) had *TP53* c.818G>A p.R273H (NGS‐UMI VAF 6.05%, dPCR VAF 5.86%), *KRAS* c.35G>T p.G12V (NGS‐UMI VAF 14.99%, dPCR VAF 10.38%), and *ERBB2* c.2524G>A p.V842I (NGS‐UMI VAF 2.53%, dPCR assay not available) (supplementary material, Table S2). In two of these three patients (patients 11 and 31), histological evidence of residual tumour was detected in endoscopic biopsies taken at *t* = 1; thus, no more blood was drawn. In one patient (patient 12), residual disease was detected earlier with ctDNA than with regular diagnostics; no histological evidence of residual tumour was found in endoscopic biopsies taken at *t* = 1. Consequently, blood was drawn, and regular diagnostics were performed at *t* = 2, which showed persistent detection of *TP53* c.586C>T p.R196* (NGS‐UMI VAF 5.41%, dPCR VAF 5.51%). Two of three patients who were ctDNA‐positive after nCRT (patients 12 and 31) had interval metastases detected on PET/CT and did not undergo surgery. One (patient 11) died of postoperative complications. All three patients were ctDNA‐positive at *t* = 0, and the degree of change in ctDNA levels from *t* = 0 to *t* = 1/*t* = 2 correlated with response to nCRT (Figure 4).

Of the 21 patients who had post‐nCRT plasma available, 14 patients had major residual disease and seven had minor residual disease in the resection specimen (Figure 2). ctDNA alone detected 3 of 14 patients with major residual disease and all of the seven patients with minor residual disease were ctDNA‐negative. This resulted in a sensitivity of 21% (95% CI 6–51) (3/14), specificity of 100% (95% CI 56–100) (7/7), PPV of 100% (95% CI 31–100) (3/3), and NPV of 39% (95% CI 18–64) (7/18). With the set of diagnostic modalities that is used in the SANO trial, 11 of 14 patients with major residual disease were detected (sensitivity of 79%, 95% CI 49–95). Of these 11 patients, nine had a positive endoscopic tumour biopsy at *t* = 1 or *t* = 2 and two had metastases detected with PET/CT at *t* = 2. With the addition of ctDNA, no additional patients were clinically identified with residual disease. All of the seven patients with minor residual disease had negative endoscopic tumour biopsies at *t* = 1 or *t* = 2, negative EUS‐FNA, and negative PET/CT scans (specificity of 100%, 95% CI 59–100). None of these patients was false‐positively identified with residual tumour by the addition of ctDNA.

### Intratumoural heterogeneity

In the group of patients with a potentially detectable *t* = 0 tissue mutation, 14 had a resection specimen available with sufficient residual vital tumour tissue for DNA isolation. In 12 of these patients (86%), the mutation identified in the *t* = 0 tumour biopsies was also identified in the resection specimen. In two patients (13%), a new, potentially detectable mutation was detected in the resection specimen. In patient 2, *KRAS* c.38G>A p.G13D was covered by the assay but not detected in the *t* = 0 tumour biopsy, while in the resection specimen, it was detected with a VAF of 31.8%. In patient 27, *TP53* c.818G>A p.R273H data were not available from NGS of the *t* = 0 tumour biopsy owing to a technical artefact. However, this mutation was identified in the resection specimen (VAF 57.5%) as well as in cfDNA at *t* = 0 (NGS‐UMI VAF 0.41%, dPCR VAF 0.47%) and *t* = 1 (NGS‐UMI VAF 0.32%, dPCR negative). Retrospectively, this mutation was also detected in the *t* = 0 tumour biopsy using Sanger sequencing but considering the aim of the study, the patient was not counted as ctDNA‐positive. In the group of patients without a potentially detectable *t* = 0 tissue mutation, one patient (patient 22) had *KRAS* c.35G>T p.G12V (VAF 8.48%) in the resection specimen, which was potentially detectable in cfDNA.

## Discussion

In the present study, we showed that ctDNA can be detected prior to treatment initiation in 38% of patients with locally advanced oesophageal cancer by correlating mutations in pre‐treatment tumour tissue with mutations in cfDNA using NGS with unique molecular identifiers and dPCR. Pre‐treatment ctDNA detection had a high PPV for disease progression before treatment or major residual disease after nCRT. The detection of ctDNA after nCRT also had a high PPV for major residual disease. However, the sensitivity and NPV of ctDNA detection before and after nCRT were low. There was no additional yield of ctDNA after nCRT compared with the current set of diagnostics that is used for detection of major residual disease.

To date, ctDNA has shown the most clinical value in the early detection of distant relapse after curative treatment and in monitoring palliative treatment [22]. Recent large studies investigating ctDNA in locally advanced oesophageal cancer have focused on post‐surgical detection of recurrence [13, 23]. The present study showed that pre‐treatment ctDNA detection may be an indicator of a poor prognosis at an early stage. Of nine pre‐treatment ctDNA‐positive patients, four had disease progression, four had major residual disease, and none had a complete response in the resection specimen. Other studies have also shown that in locally advanced and in metastatic disease, pre‐treatment ctDNA levels are correlated with treatment response and prognosis [24, 25]. Given the fact that in the present study all patients were scheduled to undergo curative treatment, the standard diagnostic work‐up may not be the optimal combination of diagnostics to select patients for either curative or palliative treatment. Pre‐treatment ctDNA may become useful in addition to the standard diagnostic work‐up for selecting patients who should undergo additional staging or even systemic therapy instead of nCRT.

The detection of ctDNA after nCRT was also an indicator of major residual disease. If ctDNA analyses after nCRT were to be added to the current set of diagnostics, however, no additional patients would be identified as having residual disease. Only Azad *et al* have also investigated post‐chemoradiotherapy ctDNA detection in locally advanced oesophageal cancer [12]. Patients underwent definitive chemoradiotherapy and thus no resection specimens were available for response assessment. Azad *et al* showed that post‐chemoradiotherapy ctDNA detection was highly predictive for distant progression but not for locoregional progression. In the present study, two of three patients in whom ctDNA was detected after nCRT had incurable disease progression prior to planned surgery. These findings suggest that locoregional residual disease may be more difficult to detect than disease progression. It is possible that small localised residues undergo less apoptosis and necrosis, presumably leading to limited shedding of ctDNA into the circulation [26]. Shedding of ctDNA may hence only increase to sufficient levels for accurate detection in cases of distant dissemination. Currently, one of the challenges of active surveillance is preventing patients from undergoing unnecessary oesophagectomy [27]. Besides patients with a complete response to nCRT, this also includes patients who develop distant metastases shortly after surgery. In the CROSS trial, 30% of patients presented with distant metastases within 2 years after nCRT and surgery [28]. Presumably, the disease had already disseminated before surgery in these patients but was still undetectable on preoperative PET/CT. The detection of disseminated disease by increasing levels of ctDNA may precede the detection of overt metastases on PET/CT [12, 29]. This lead time of ctDNA detection may help to detect subclinical metastases before surgery, which could become useful in sparing patients from undergoing unnecessary oesophagectomy.

In other types of cancer, high PPVs with low NPVs have been reported for the detection of locoregional residual disease in resection specimens. A side‐study of the CRITICS trial showed that the PPV for detection of substantial locoregional residual disease after preoperative chemotherapy in patients with gastric cancer was 100%, while the NPV was only 26% [30]. For detection of any locoregional residual disease, a PPV of 88% with an NPV of 50% was found after neoadjuvant chemotherapy for triple‐negative breast cancer in the Q‐CROC trial, and a PPV of 83% with an NPV of 11% was found after total neoadjuvant treatment for rectal cancer in the GEMCAD 1402 trial [31, 32]. These results emphasise the challenges of using ctDNA as the sole biomarker for locoregional residual disease.

The present study has several strengths. To our knowledge, this is the first study on locally advanced oesophageal cancer that compared the detection of ctDNA with findings in post‐nCRT endoscopic biopsies and resection specimens. Since the detection of ctDNA is most reliable when tumour‐specific mutations in pre‐treatment tumour tissue are known, we only considered patients to be ctDNA‐positive if tissue mutations could be matched with mutations in cfDNA. In this way, other methods to control for clonal haematopoiesis such as leukocyte sequencing are not necessary. For ultrasensitive detection, we used NGS‐UMI as well as dPCR whenever possible [33]. NGS‐UMI has a limit of detection VAF of >0.1%, whereas dPCR can measure down to >0.01%. In contrast to dPCR, however, NGS‐UMI can also detect novel mutations that develop during or after treatment.

Several limitations should be mentioned. Matching tissue mutations with cfDNA mutations introduced a risk of false‐negative findings because of spatial and/or temporal heterogeneity (i.e. cfDNA mutations cannot be matched with subclonal tissue mutations). Since most known driver mutations are homogeneously present, this risk seems to be limited [34]. This was confirmed in the present study, showing that *t* = 0 tissue mutations could be confirmed in the resection specimen in 86% of patients. Also, a risk of false‐negative findings may have been introduced by the lower cfDNA input for NGS‐UMI than that for dPCR. To decrease false‐negative findings in future studies, cfDNA input can be increased by drawing more blood. In addition, technical improvements, such as the use of target methylation analysis of cfDNA, could further increase sensitivity [35]. Furthermore, this study comprised a small sample size and was designed to explore the feasibility of using ctDNA in identifying residual disease. The small sample size limits us in drawing conclusions regarding the true discriminative power of ctDNA in who should or should not undergo surgery after nCRT.

In conclusion, our data suggest that pre‐treatment as well as post‐nCRT ctDNA detection may become useful in identifying patients at high risk of disease progression. How ctDNA can be best utilised to discriminate these patients from patients with (primary or residual) resectable locoregional disease requires further investigation in a diagnostic trial. The addition of ctDNA as a non‐invasive biomarker to the current set of diagnostic modalities may not improve the detection of residual or recurrent disease during active surveillance after nCRT. The low sensitivities of ctDNA as a sole modality indicate that ctDNA may also not be able to replace current diagnostics and indicate challenges for further research into the role of ctDNA as a sole modality for detecting smaller amounts of locoregional residual disease after nCRT.

## Author contributions statement

All the authors contributed to the design of the study and data collection. BME, MPHMJ, BJN, PNA and WNMD analysed the data. BME and MPHMJ wrote the first draft of this report. All the authors contributed to data interpretation and reviewed and approved the final version of the manuscript.

## Supporting information


**Figure S1.** Correlation of baseline tumour characteristics with variant allele frequency (VAF) of nine pre‐treatment ctDNA‐positive patients
**Table S1**. Input information of the analysed tissue and plasma samples
**Table S2**. Mutations identified in baseline (*t* = 0) tissue that were potentially detectable in circulating cell‐free DNA (cfDNA) with corresponding findings in cfDNA at the baseline (*t* = 0) and 6 weeks (*t* = 1) and 12 weeks (*t* = 2) after neoadjuvant chemoradiotherapy

## Data Availability

The human sequence data generated in this study are not publicly available because of patient privacy requirements but are available upon reasonable request from the corresponding author. Other data generated in this study are available in the article and its supplementary data files.
