# Supplementary material for: Detection of circulating tumour DNA after neoadjuvant chemoradiotherapy in patients with locally advanced oesophageal cancer
Source: J Pathol. 2022 Oct 31;259(1):35–45. doi: 10.1002/path.6016 (PMC10092085; doi:10.1002/path.6016)
Supplement: Supplementary file 1 — Figure S1. Correlation of baseline tumour characteristics with variant allele frequency (VAF) of nine pre‐treatment ctDNA‐positive patients Table S1. Input information of the analysed tissue and plasma samples Table S2. Mutations identified in baseline (t = 0) tissue that were potentially detectable in circulating cell‐free DNA (cfDNA) with corresponding findings in cfDNA at the baseline (t = 0) and 6 weeks (t = 1) and 12 weeks (t = 2) after neoadjuvant chemoradiotherapy [file PATH-259-35-s001.pdf]

Detection of circulating tumour DNA after neoadjuvant chemoradiotherapy in patients with locally advanced oesophageal cancer

BM Eyck *et al.* *J Pathol* https://doi.org/10.1002/path.6016

Supplementary Figure S1

Supplementary Tables S1–S2

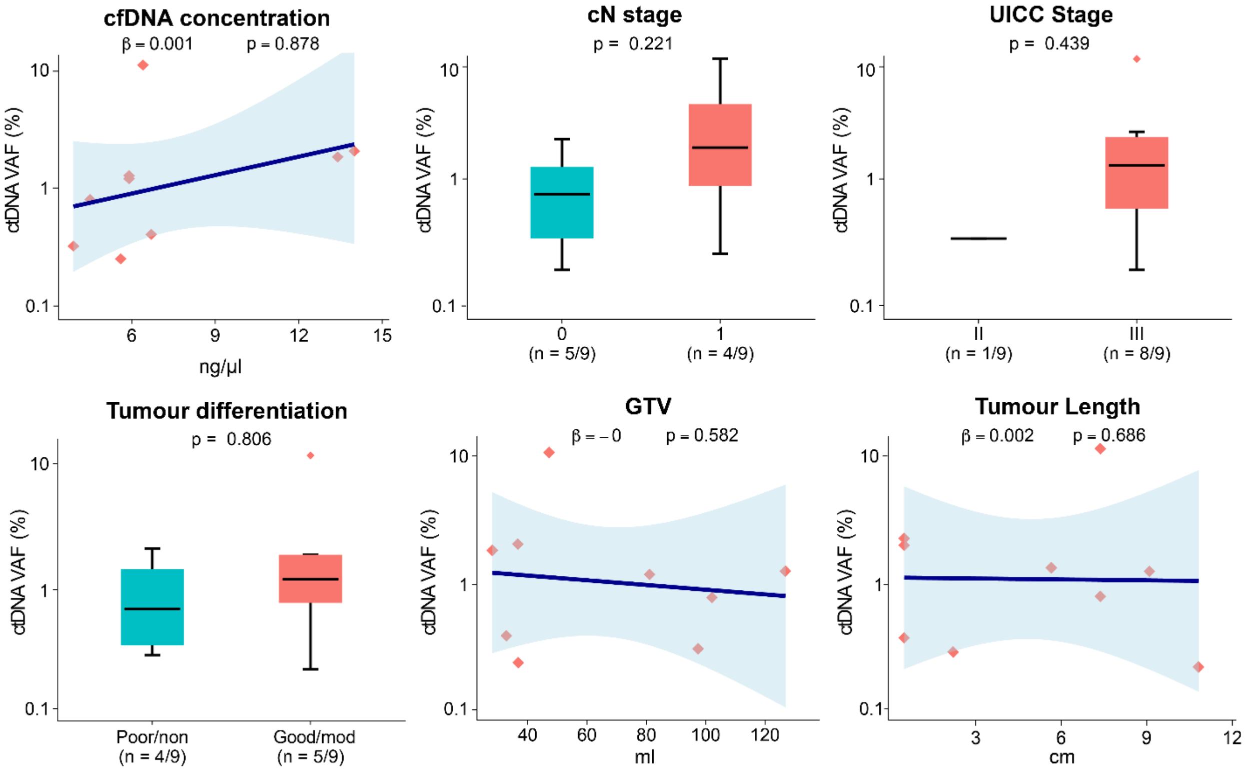

Figure S1. Correlation of baseline tumour characteristics with variant allele frequency (VAF) of nine pre-treatment ctDNA-positive patients. Patients for whom data were missing were excluded from the analysis. cN, clinical nodal; UICC, Union for International Cancer Control; GTV, gross tumour volume.

Table S1. Input information of the analysed tissue and plasma samples.

	Tissue samples for NGS				cfDNA samples for NGS-UMI			cfDNA samples for dPCR		
	t = 0 tumour biopsies		Resection specimens		*t* = 0	*t* = 1	*t* = 2	*t* = 0	*t* = 1	*t* = 2
Study ID	Tumour area (%)	DNA concentration (ng/µl)	Tumour area (%)	DNA concentration (ng/µl)	DNA input (ng)	DNA input (ng)	DNA input (ng)	DNA input (ng)	DNA input (ng)	DNA input (ng)
1	60%	1.57	NTR	–	20.0	21.0	21.0	25.7	39.1	38.7
2	30%	1.70	70%	1.85	20.0	15.0	10.0	–	–	–
3	90%	1.06	NTR	–	14.0	20.0	21.0	1.1	5.5	6.9
4	80%	4.16	60%	1.03	21.5	20.0	14.0	–	–	–
5	50%	3.98	NR	–	3.8	15.0	3.8	15.7	18.9	29.5
6	50%	1.70	70%	13.50	3.8	10.2	20.0	–	–	–
7	40%	1.40	40–50%	4.10	4.0	5.1	–	9.6	12.5	–
8	60%	7.10	70–80%	15.30	4.0	18.2	5.0	8.4	24.8	28.3
9	40%	1.80	NR	–	4.0	–	–	–	–	–
10	70%	10.80	70%	15.80	4.0	31.8	–	9.3	17.6	–
11	60%	5.50	80%	15.50	4.0	5.9	–	–	–	–
12	70%	16.10	NR	–	4.0	4.5	N/A	19.0	29.8	26.4
13	80%	9.31	90%	17.60	–	–	–	–	–	–
14	80%	3.90	NR	–	–	–	–	–	–	–
15	80%	6.92	NR	–	4.0	–	–	–	–	–
16	60%	12.10	NR	–	–	–	–	–	–	–
17	50%	4.02	70%	0.96	20.0	20.0	–	–	–	–
18	60%	7.31	NTR	–	–	–	–	14.8	5.9	4.8
19	N/A	2.79	NTR	–	15.3	23.0	21.0	–	–	–
20	N/A	N/A	80%	5.34	N/A	21.0	–	–	–	–
21	60%	20.70	NTR	–	–	–	–	–	–	–
22	70%	5.04	80%	6.91	–	–	–	–	–	–
23	70%	9.65	NTR	–	–	–	–	–	–	–
24	50%	0.76	70%	0.48	20.7	20.0	20.0	0.0	3.5	21.3
25	70%	2.89	95%	10.10	20.8	20.0	21.0	19.1	20.2	2.9
26	70%	1.15	60%	1.42	–	–	–	–	–	–
27	70%	0.91	60%	13.10	15.4	24.0	–	–	–	–
28	80%	4.05	60%	2.30	4.0	3.3	1.0	9.3	13.0	14.9
29	70%	12.30	60%	1.75	3.9	4.9	–	6.6	13.3	–
30	80%	5.90	NR	–	3.9	–	–	20.9	–	–
31	70%	7.10	NR	–	3.9	130.2	N/A	13.3	21.7	N/A

NGS, next-generation sequencing; cfDNA, circulating cell-free DNA; NGS-UMI, next-generation sequencing with unique molecular identifiers; dPCR, digital polymerase chain reaction; NTR, no tumour residue in resection specimen; NR, no resection performed; ND, no data; N/A, not available.

Table S2. Mutations identified in baseline (*t* = 0) tissue that were potentially detectable in circulating cell-free DNA (cfDNA) with corresponding findings in cfDNA at the baseline (*t* = 0) and 6 weeks (*t* = 1) and 12 weeks (*t* = 2) after neoadjuvant chemoradiotherapy. Findings shown in bold were considered to be ctDNA-positive.

	***t* = 0 tissue**						***t* = 0 cfDNA**						***t* = 1 cfDNA**						***t* = 2 cfDNA**					
	**NGS**				**cfDNA analysis**	**NGS-UMI**			**dPCR**			**NGS-UMI**			**dPCR**			**NGS-UMI**			**dPCR**		
**ID**	**Gene**	**CDS mutation**	**AA mutation**	**VAF (%)**		**Mol cov**	**Mut mol**	**VAF (%)**	**WT cop**	**Mut cop**	**VAF (%)**	**Mol cov**	**Mut mol**	**VAF (%)**	**WT cop**	**Mut cop**	**VAF (%)**	**Mol cov**	**Mut mol**	**VAF (%)**	**WT cop**	**Mut cop**	**VAF (%)**
1	*TP53*	c.524G>A	p.R175H	69.2%	O/P	2968	1	0.03%	5558	0	0.00%	5091	2	0.04%	13980	0	0.00%	4433	0	0.00%	11922	0	0.00%
2	*TP53*	c.527G>T	p.C176F	45.1%	O	1248	0	0.00%	–	–	–	2637	0	0.00%	–	–	–	1391	0	0.00%	–	–	–
3	*TP53*	c.742C>T	p.R248W	41.9%	O/P	1377	0	0.00%	1168	0	0.00%	2598	0	0.00%	3606	0	0.00%	N/A	N/A	N/A	4340	0	0.00%
4	*TP53*	c.709_733dup	p.G245Dfs*3	48.2%	O	1563	0	0.00%	–	–	–	3457	0	0.00%	–	–	–	2532	0	0.00%	–	–	–
5	*TP53*	c.455C>T	p.P152L	64.1%	O/P	1536	1	0.07%	2733	0	0.00%	2259	0	0.00%	0	0	0.00%	3362	2	0.06%	0	0	0.00%
6	*TP53*	c.659A>G	p.Y220C	68.9%	O	888	0	0.00%	–	–	–	1513	0	0.00%	–	–	–	1802	0	0.00%	–	–	–
7	*AKT1*	c.49G>A	p.E17K	55.3%	O	1162	0	0.00%	–	–	–	791	0	0.00%	–	–	–	–	–	–	–	–	–
7	*TP53*	c.524G>A	p.R175H	50.5%	O/P	1314	0	0.00%	2654	0	0.00%	950	0	0.00%	1075	0	0.00%	–	–	–	–	–	–
8	*TP53*	c.818G>A	p.R273H	54.3%	O/P	1094	1	0.09%	1282	4	0.30%	1886	0	0.00%	4068	0	0.00%	568	0	0.00%	416	0	0.00%
9	*TP53*	c.637C>T	p.R213*	51.3%	O	696	0	0.00%	–	–	–	–	–	–	–	–	–	–	–	–	–	–	–
10	*TP53*	c.817C>T	p.R273C	27.0%	O/P	707	2	0.28%	2117	0	0.00%	569	0	0.00%	87	0	0.00%	–	–	–	–	–	–
11	*TP53*	c.764_766del	p.I255del	58.1%	O	**1214**	**3**	**0.25%**	–	–	–	**1574**	**6**	**0.38%**	–	–	–	–	–	–	–	–	–
12	*TP53*	c.586C>T	p.R196*	44.2%	O/P	1032	2	0.19%	**5113**	**16**	**0.32%**	834	0	0.00%	**7235**	**20**	**0.28%**	**795**	**43**	**5.41%**	**5135**	**300**	**5.51%**
15	*APC*	c.4391_4394del	p.E1464Vfs*8	68.3%	O	**1728**	**22**	**1.27%**	–	–	–	–	–	–	–	–	–	–	–	–	–	–	–
17	*TP53*	c.413C>T	p.A138V	60.2%	O	2663	0	0.00%	–	–	–	3261	0	0.00%	–	–	–	–	–	–	–	–	–
18	*TP53*	c.916C>T	p.R306*	11.2%	P	–	–	–	5438	0	0.00%	–	–	–	3800	4	0.10%	–	–	–	4550	0	0.00%
19	*KRAS*	c.182A>T	p.Q61L	23.7%	O	1658	0	0.00%	–	–	–	2313	0	0.00%	–	–	–	1832	0	0.00%	–	–	–
20	*TP53*	c.578A>C	p.H193P	44.0%	O	1080	0	0.00%	–	–	–	2012	0	0.00%	–	–	–	–	–	–	–	–	–
24	*TP53*	c.818G>A	p.R273H	41.4%	O/P	**1710**	**7**	**0.40%**	N/A	N/A	N/A	2550	0	0.00%	1417	2	0.14%	2855	0	0.00%	762	0	0.00%
25	*TP53*	c.524G>A	p.R175H	79.1%	O/P	**2016**	**24**	**1.20%**	**5273**	**27**	**0.51%**	3579	0	0.00%	9733	0	0.00%	3442	0	0.00%	2296	0	0.00%
27	*TP53*	c.772G>A	p.E258K	48.0%	O	3259	0	0.00%	–	–	–	3760	0	0.00%	–	–	–	–	–	–	–	–	–
28	*TP53*	c.524G>A	p.R175H	89.8%	O/P	**1435**	**141**	**9.83%**	**2133**	**272**	**11.32%**	722	0	0.00%	801	0	0.00%	673	0	0.00%	2425	0	0.00%
29	*TP53*	c.524G>A	p.R175H	61.6%	O/P	**1149**	**4**	**0.35%**	**1260**	**10**	**0.79%**	828	0	0.00%	3758	0	0.00%	–	–	–	–	–	–
30	*TP53*	c.733G>A	p.G245S	57.5%	O/P	**4466**	**70**	**1.57%**	**5758**	**122**	**2.07%**	–	–	–	–	–	–	–	–	–	–	–	–
31	*TP53*	c.818G>A	p.R273H	65.3%	O/P	**2764**	**47**	**1.70%**	**2397**	**45**	**1.85%**	**5408**	**327**	**6.05%**	**6199**	**386**	**5.86%**	–	–	–	–	–	–
31	*ERBB2*	c.2524G>A	p.V842I	36.2%	O	**3730**	**21**	**0.56%**	–	–	–	**7153**	**181**	**2.53%**	–	–	–	–	–	–	–	–	–
31	*KRAS*	c.34G>T	p.G12C	19.9%	O/P	3457	0	0.00%	4446	0	0.00%	12010	0	0.00%	18301	0	0.00%	–	–	–	–	–	–
31	*KRAS*	c.35G>T	p.G12V	15.9%	O/P	**5212**	**95**	**2.75%**	**5673**	**96**	**1.66%**	**8393**	**1258**	**14.99%**	**19384**	**2246**	**10.38%**	–	–	–	–	–	–

NGS, next-generation sequencing; NGS-UMI, next-generation sequencing with unique molecular identifiers; dPCR, digital polymerase chain reaction; Resec spec, resection specimen; CDS, coding DNA sequence; AA, amino acid; VAF, variant allele frequency; Mol cov, molecular coverage; Mut mol, mutant molecules; WT cop, wild-type copies; Mut cop, mutant copies; O, Oncomine NGS; P, digital PCR; N/A, not available.
